# Supplementary material for: Identification of Breathing Patterns through EEG Signal Analysis Using Machine Learning
Source: Brain Sci. 2021 Feb 26;11(3):293. doi: 10.3390/brainsci11030293 (PMC7996914; doi:10.3390/brainsci11030293)
Supplement: Supplementary file 1 [file brainsci-11-00293-s001.pdf]

Supplementary Materials: Identification of breathing patterns through EEG signal analysis using machine learning

Yong-Gi Hong [1], Hang-Keun Kim [1,2*], Young-Don Son [1,2] and Chang-Ki Kang [1,3*]

Table S1. SpO2 difference between groups.

(A) ANOVA for 1_back (1B) task group

	Sum of Squares	df	Mean Square	F	p
group_1B	7.8839	2	3.942	11.118	< .001
Residuals	19.1459	54	0.355		

Comparison		Mean Difference	SE	t	p_{tukey}
1B_M	1B_N	0.062	0.193	0.321	0.945
-	1B_O2	-0.756	0.193	-3.914	**< .001**
1B_N	1B_O2	-0.818	0.193	-4.235	**< .001**

	SpO2		95% Confidence Interval	
	Mean	SE	Lower	Upper
1B_M	97.692	0.137	97.418	97.966
1B_N	97.630	0.137	97.356	97.904
1B_O2	98.448	0.137	98.174	98.722

(B) ANOVA for 2_back (2B) task group

	Sum of Squares	df	Mean Square	F	p
group_2B	6.216	2	3.108	9.286	< .001
Residuals	18.076	54	0.335		

Comparison		Mean Difference	SE	t	p_{tukey}
2B_M	2B_N	0.198	0.188	1.057	0.545
-	2B_O2	-0.580	0.188	-3.090	**0.009**
2B_N	2B_O2	-0.778	0.188	-4.147	**< .001**

	SpO2		95% Confidence Interval	
	Mean	SE	Lower	Upper
2B_M	97.898	0.133	97.632	98.164
2B_N	97.700	0.133	97.434	97.966
2B_O2	98.478	0.133	98.212	98.744

(C) ANOVA for Resting group

	Sum of Squares	df	Mean Square	F	p
group_Rest	25.774	5	5.155	10.462	< .001
Residuals	51.241	104	0.493		

Comparison		Mean Difference	SE	t	p_{tukey}
Rest_CE_M	Rest_CE_N	-0.165	0.228	-0.725	0.978
-	Rest_CE_O2	-1.064	0.228	-4.673	**< .001**
-	Rest_OE_M	-0.218	0.234	-0.931	0.938
-	Rest_OE_N	-0.218	0.231	-0.946	0.933
-	Rest_OE_O2	-1.249	0.231	-5.408	**< .001**
Rest_CE_N	Rest_CE_O2	-0.899	0.228	-3.948	**0.002**
-	Rest_OE_M	-0.053	0.234	-0.226	1
-	Rest_OE_N	-0.053	0.231	-0.231	1
-	Rest_OE_O2	-1.084	0.231	-4.693	**< .001**
Rest_CE_O2	Rest_OE_M	0.846	0.234	3.610	**0.006**
-	Rest_OE_N	0.846	0.231	3.663	**0.005**
-	Rest_OE_O2	-0.184	0.231	-0.799	0.967
Rest_OE_M	Rest_OE_N	0.000	0.237	-0.001	1
-	Rest_OE_O2	-1.031	0.237	-4.341	**< .001**
Rest_OE_N	Rest_OE_O2	-1.030	0.234	-4.403	**< .001**

	SpO2		95% Confidence Interval	
	Mean	SE	Lower	Upper
Rest_CE_M	97.224	0.161	96.905	97.544
Rest_CE_N	97.390	0.161	97.070	97.709
Rest_CE_O2	98.289	0.161	97.969	98.608
Rest_OE_M	97.443	0.170	97.105	97.780
Rest_OE_N	97.443	0.165	97.115	97.771
Rest_OE_O2	98.473	0.165	98.145	98.801

Table S2. Behavioral result of working memory (WM) accuracy between groups.

ANCOVA - WM Accuracy

	Sum of Squares	df	Mean Square	F	p
Group	165.84	5	33.168	0.585	0.711
Resp_Time	1892.27	1	1892.272	33.377	< 0.001
Residuals	6406.34	113	56.693		

	WM Accuracy		95% Confidence Interval	
**Group**	**Mean ± SE**		**Lower**	**Upper**
1B_N	92.718	± 1.714	89.323	96.113
2B_N	92.487	± 1.690	89.138	95.835
1B_M	93.777	± 1.717	90.375	97.179
2B_M	93.857	± 1.706	90.478	97.236
1B_O2	90.455	± 1.686	87.114	93.796
2B_O2	93.623	± 1.716	90.223	97.023

Table S3. The absolute values of the five important upper positive and negative weight vector channels. Note that the ranks were the same when gamma wave excluded.

(A) The ranking of significant waves and channels.

**With Gamma Wave**		**Without Gamma Wave**	
Wave / location	Absolute value *	Wave / location	Absolute value
Beta / FC6	1.079	Beta / FC6	1.400
Beta / AF4	0.649	Beta / AF4	1.010
Beta / P7	0.595	Beta / O1	0.657
Beta / O2	0.423	Beta / AF3	0.646
Theta / C4	0.369	Theta / C4	0.630
Beta / FC5	0.514	Beta / F4	0.571
Beta / Oz	0.530	Beta / AF3	0.611
Beta / F3	0.541	Beta / P3	0.733
Theta / CP6	0.546	Theta / F7	1.092
Beta / CP6	0.776	Beta / FC5	1.169

* The values were obtained when gamma waves were ignored for ranking.

(B) The ranking of LDA coefficients.

**Sum of Squares**	**LDA coefficient (Absolute Sum)**	
	**With Gamma Wave**	**Without Gamma Wave**
Gamma	32.203	
Beta	9.232	12.731
Theta	5.036	7.858
Alpha	4.697	4.412
Delta	3.142	3.862
